# Using pulse oximetry waveforms to detect coarctation of the aorta

**DOI:** 10.1186/s12938-020-00775-2

**Published:** 2020-05-14

**Authors:** Matthew W. Sorensen, Ismail Sadiq, Gari D. Clifford, Kevin O. Maher, Matthew E. Oster

**Affiliations:** 1 Division of Cardiology, Pediatric Cardiology, Children’s Healthcare of Atlanta, 1405 Clifton Rd, Atlanta, GA 30322 USA; 2 Department of Pediatrics, Emory University School of Medicine, Atlanta, GA USA; 3 Department of Biomedical Informatics, Emory University School of Medicine, Woodruff Memorial Research Building, 101 Woodruff Circle, 4th Floor East, Atlanta, GA 30322 USA; 4 Department of Electrical Engineering, Georgia Institute of Technology, Atlanta, GA USA; 5 Department of Biomedical Engineering, Georgia Institute of Technology and Emory University, Atlanta, GA USA

**Keywords:** Coarctation, Critical congenital heart disease screening, Pulse oximetry, Waveform analysis

## Abstract

**Background:**

Coarctation of the aorta is a common form of critical congenital heart disease that remains challenging to diagnose prior to clinical deterioration. Despite current screening methods, infants with coarctation may present with life-threatening cardiogenic shock requiring urgent hospitalization and intervention. We sought to improve critical congenital heart disease screening by using a novel pulse oximetry waveform analysis, specifically focused on detection of coarctation of the aorta.

**Methods and results:**

Over a 2-year period, we obtained pulse oximetry waveform data on 18 neonates with coarctation of the aorta and 18 age-matched controls hospitalized in the cardiac intensive care unit at Children’s Healthcare of Atlanta. Patients with coarctation were receiving prostaglandin E1 and had a patent ductus arteriosus. By analyzing discrete features in the waveforms, we identified statistically significant differences in the maximum rate of fall between patients with and without coarctation. This was accentuated when comparing the difference between the upper and lower extremities, with the lower extremities having a shallow slope angle when a coarctation was present (*p*-value 0.001). Postoperatively, there were still differences in the maximum rate of fall between the repaired coarctation patients and controls; however, these differences normalized when compared with the same individual’s upper vs. lower extremities. Coarctation patients compared to themselves (preoperatively and postoperatively), demonstrated waveform differences between upper and lower extremities that were significantly reduced after successful surgery (*p*-value 0.028). This screening algorithm had an accuracy of detection of 72% with 0.61 sensitivity and 0.94 specificity.

**Conclusions:**

We were able to identify specific features in pulse oximetry waveforms that were able to accurately identify patients with coarctation and further demonstrated that these changes normalized after surgical repair. Pulse oximetry screening for congenital heart disease in neonates may thus be improved by including waveform analysis, aiming to identify coarctation of the aorta prior to critical illness. Further large-scale testing is required to validate this screening model among patients in a newborn nursery setting who are low risk for having coarctation.

## Background

**Fig. 1:**
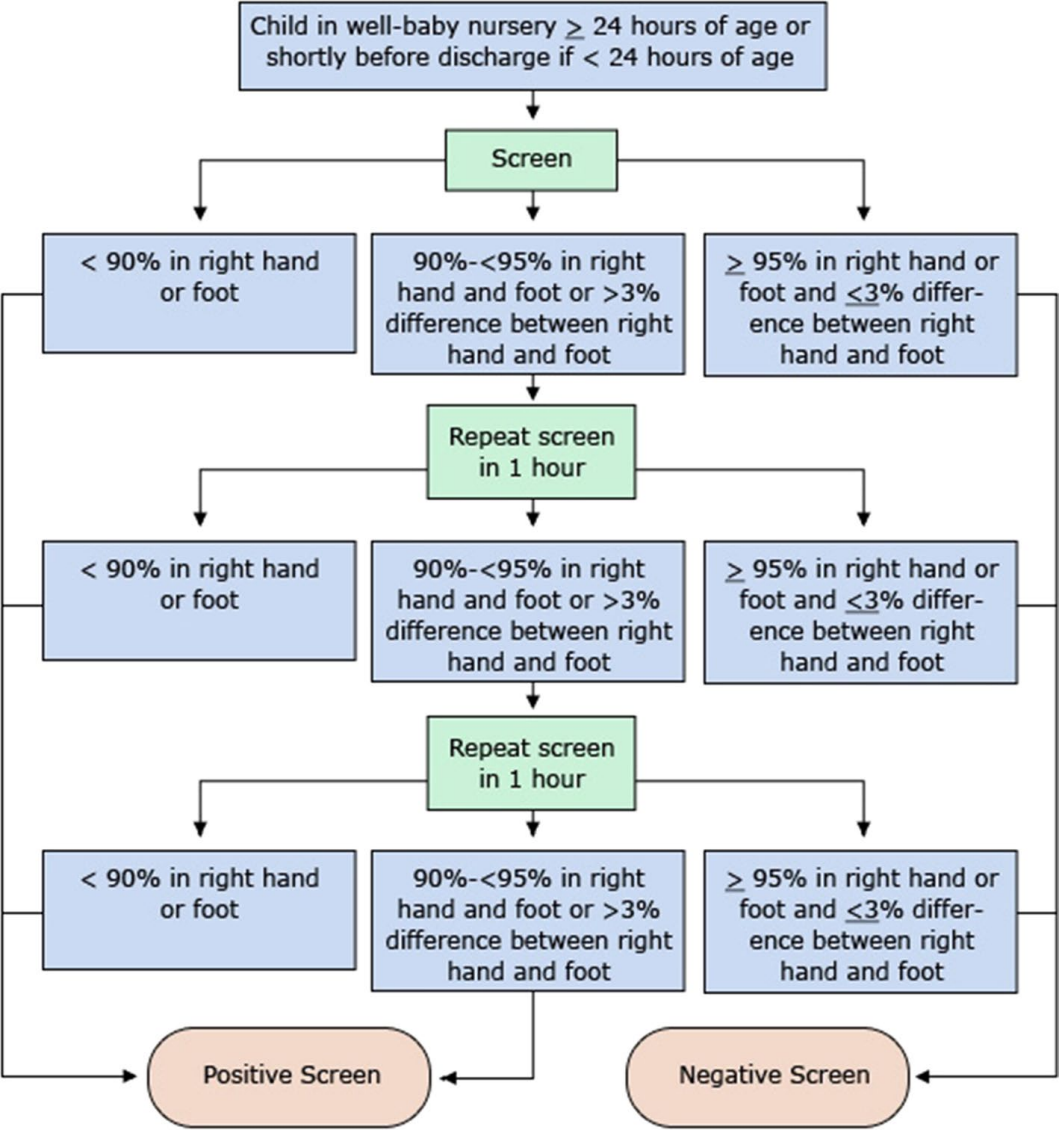
Example of neonatal pulse oximetry screening algorithm. Reprinted from the public domain at the CDC’s *Congenital Heart Defects Information for Healthcare Providers* (https://www.cdc.gov/ncbddd/heartdefects/hcp.html) [18]. Note that an indeterminate zone merits repeated screening in order to attempt to minimize false positives

**Fig. 2:**
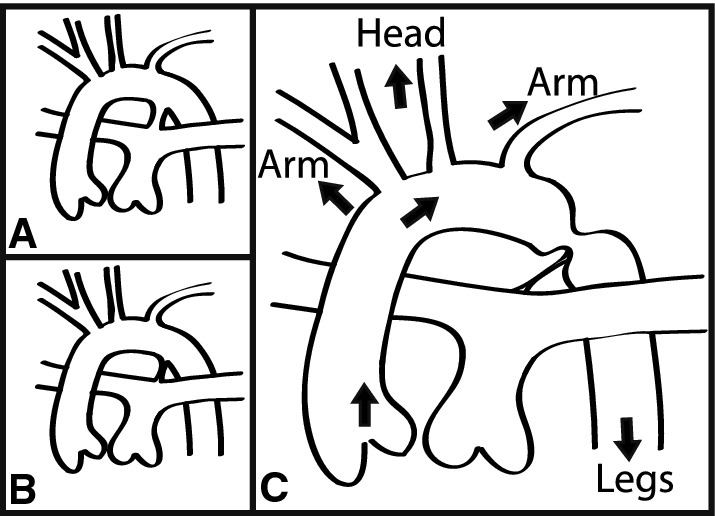
Schematic of aortic arch with and without coarctation. **a** Shows normal fetal circulation with a patent ductus arteriosus (PDA) connecting the pulmonary artery to the aorta. **b** Shows normal constriction of the PDA in post-natal circulation. **c** Shows the most common location for aortic coarctation. Note the head, neck and upper extremity vessels branch off the aorta proximal to the most common location to have coarctation of the aorta. The arteries that perfuse the legs are located more distal from the coarctation. Thus, waveform analysis is able to identify pulse oximetry signal characteristics both proximal and distal to site of coarctation

Coarctation of the aorta (COA) is one of the most common lesions of congenital heart disease, accounting for approximately 7% of all cases [1], 4 per 10,000 births [2] or approximately 1600 newborns per year. Despite this prevalence, it frequently eludes detection in both the prenatal and neonatal periods [3]. Current neonatal screening detects hypoxemia in newborns using pulse oximetry analysis from different extremities in an algorithmic approach (see Fig. 1) in order to identify critical congenital heart disease (CCHD) [4] before hospital discharge. It is important to evaluate the PPG waveform from extremities supplied by branch vessels both proximal and distal to the patent ductus arteriosus (PDA) and potential coarctation (see Fig. 2). This method of screening does well to detect cyanotic mixing lesions using only numeric values of oxygen saturation. Of the 12 CCHDs, COA is the most common, yet has the worst false-positive rate and only 46% sensitivity with current screening methods [5–7]. Even with prenatal ultrasound and pulse oximetry screening the majority (53–62%) of COA cases are late diagnoses [6, 7] and many such infants present to medical care only once they are in life-threatening extremis.

Current newborn screens are inadequate to detect COA, but repurposing existing technology may allow for more timely diagnosis by analyzing waveform data in place of solely using integer values of arterial oxygen saturation. Photoplethysmography (PPG) is routinely seen as a live waveform on pulse oximetry machines that is a composite of multiple waves which have shown promise in detecting changes in peripheral perfusion, blood pressure and local vasomotor tone [8]. Comparing waveform analysis with current non-invasive blood pressure monitoring, pulse oximetry technology may be equal to if not better than current non-invasive techniques [8]. The clinical utility of analyzing PPG waveforms has been demonstrated in newborns with COA in order to quantify physical exam characteristics such as diminished lower extremity pulses and pulse arrival time between extremities [9]. Additional studies have shown promise in PPG signals identifying the presence of significant patent ductus arteriosus in premature infants [10] as well as PPG may be a useful marker to trend cardiac output and stroke volume [11]. The purpose of this study was to incorporate PPG waveform data to develop an algorithm that can increase the sensitivity of detection of coarctation of the aorta as compared to existing methods. We hypothesized that an algorithmic approach to analyze PPG waveforms would increase the sensitivity of detection of coarctation of the aorta compared to existing methods.

## Results

We identified a cohort of 18 patients with COA requiring surgical intervention as neonates and matched them with 18 control patients who met inclusion and exclusion criteria listed in the Methods section. See Table 1 for patient group characteristics. There were no significant differences in gender or ethnicity. Gender did not affect the upper or lower extremity PPG waveforms as the underlying vascular anatomy is the same for monitoring PPG on the digits or distal extremities. Of the control group, 9 patients (50%) had some degree of anomalous pulmonary venous return, 4 (22%) had neonatal arrhythmias, 2 (11%) had valvar abnormalities and 3 others had cardiomyopathy, pulmonary hypertension and a pericardial tumor (representing 6% each).

**Table 1:** Patient cohort characteristics

	Control		Coarctation	
Sex				
Male	11	61%	9	50%
Female	7	39%	9	50%
Race/ethnicity				
African American	8	44%	5	28%
White	8	44%	13	72%
Hispanic	2	11%	3	17%
Other/unanswered	2	11%	0	0%
Diagnoses of control group				
Pulmonary vein	9	50%		
Arrhythmia	4	22%		
Valvar abnormality	2	11%		
Cardiomyopathy	1	6%		
Pulmonary hypertension	1	6%		
Tumor	1	6%		

Here we report the demographic information of each cohort and report the primary diagnosis resulting in neonatal admission of the control group to the cardiac intensive care unit

### Preoperative COA vs. unaffected controls

**Fig. 3:**
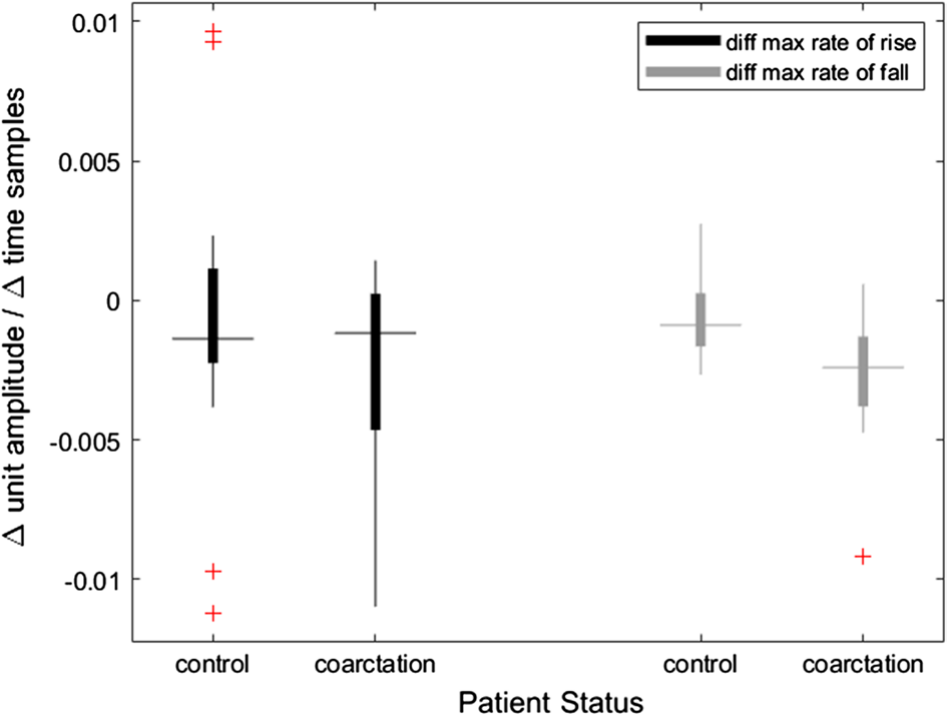
Preoperative differences in maximum rates of rise and fall. The *Y*-axis represents a normalized slope (waveform amplitude divided by time) where a greater difference means there was a noticeable difference when comparing upper vs lower extremity waveforms. There was no significant difference in rate of rise between groups (*p* value 0.54), but there was a significant difference of rate of fall between the control and the coarctation group (*p* value 0.004)

There was a near-linear association between the average rate of rise and fall compared to heart rate (Additional file 1: Figure S1). Presurgical waveform characteristics between COA and control patients were calculated and shown in Table 2. The two characteristics that were significant (*p*-value < 0.01) were the maximum rate of fall (MRF) in the lower extremity and the difference between the MRF of the upper extremity minus the lower extremity (∆MRF on Table 2 and Fig. 3).

**Table 2:** Preoperative waveform analysis for upper and lower extremities and their differences

Preop feature	Control	Coarctation	*p* value
	Median [Q1, Q3]		
ARR_u	4.66 [4.38, 5.07]	4.52 [4.41, 4.88]	0.16
ARF_u	− 1.72 [− 1.87, − 1.53]	− 1.67 [− 1.75, − 1.53]	0.96
MRR_u	5.05 [4.71, 5.46]	4.98 [4.76, 5.26]	0.24
MRF_u	− 2.63 [− 2.81, − 2.47]	− 2.64 [− 2.73, − 2.51]	0.6
ARR_l	4.9 [4.42, 5.32]	4.9 [4.47, 5.31]	0.91
ARF_l	− 1.53 [− 1.65, − 1.41]	− 1.49 [− 1.59, − 1.35]	0.38
MRR_l	5.27 [4.77, 5.68]	5.32 [4.87, 5.7]	0.84
MRF_l**	− 2.6 [− 2.67, − 2.52]	− 2.34 [− 2.5, − 2.3]	0.009
∆ARR	− 0.22 [− 0.52, − 0.13]	− 0.12 [− 0.45, 0.02]	0.5
∆ARF	− 0.12 [− 0.31, − 0.02]	− 0.2 [− 0.26, − 0.01]	0.22
∆MRR	− 0.22 [− 0.44, − 0.11]	− 0.12 [− 0.46, 0.02]	0.54
∆MRF**	− 0.07 [− 0.2, 0.08]	− 0.24 [− 0.38, − 0.13]	0.001

Units for slope are normalized amplitude/time and values are reported as the median, 1st and 3rd quartiles of PPG features. Subscripts indicate upper (u) vs lower (l) extremity acquisition site. The ∆ indicates the difference between the upper and lower extremity for that feature. Features with statistical significance between the 2 groups are marked as **p value < 0.01

*ARR* average rate of rise, *ARF* average rate of fall, *MRR* maximum rate of rise, *MRF* maximum rate of fall, *COA* coarctation of the aorta, *Q1* 1st quartile, *Q3* 3rd quartile

### Postoperative COA vs. unaffected controls

**Fig. 4:**
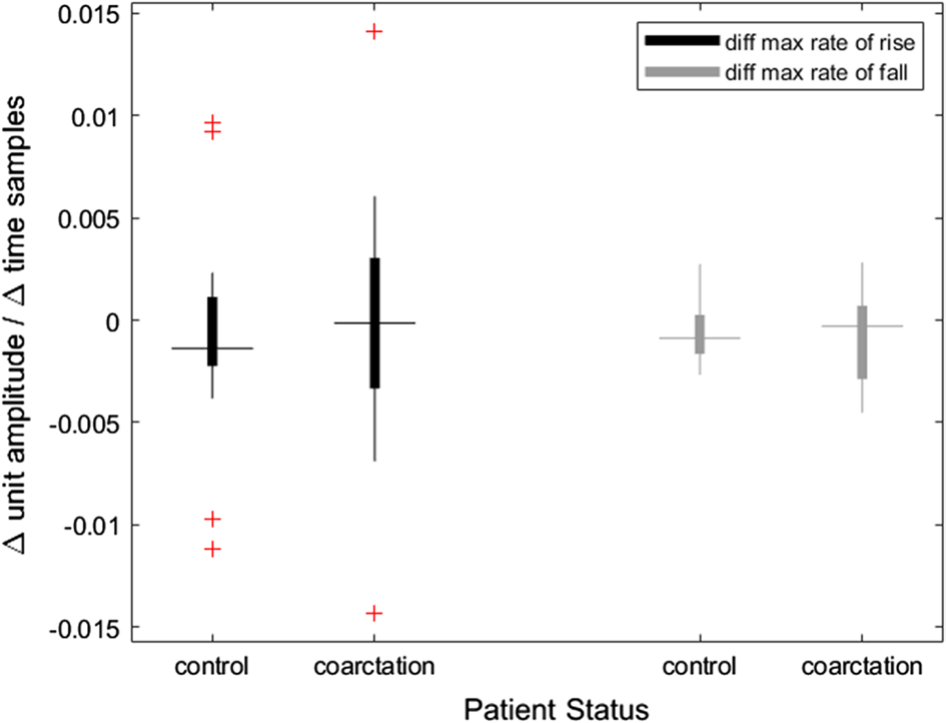
Postoperative differences in maximum rates of rise and fall. The *Y*-axis represents a normalized slope (waveform amplitude divided by time). There was no significant difference between groups in rate of rise (*p* value 0.61) or fall (*p* value 0.93) following surgical repair of coarctation of the aorta

Of the 18 patients with COA, one patient did not have upper extremity PPG data recorded postoperatively, therefore they were withheld from the postop statistical analysis. Table 3 shows the same waveform characteristics evaluated postoperatively. Here, we note statistical significance in the MRF in both the upper and lower extremities but not in the ∆MRF, indicating that the difference normalized after surgical correction of the COA (see Fig. 4).

**Table 3:** Postoperative waveform analysis for upper and lower extremities and their differences

Postop feature	Control	Coarctation	*p* value
	Median [Q1, Q3]		
ARR_u	4.66 [4.38, 5.07]	4.68 [4.44, 4.86]	0.46
ARF_u	− 1.72 [− 1.87, − 1.53]	− 1.65 [− 1.79, − 1.54]	0.59
MRR_u	5.05 [4.71, 5.46]	5.04 [4.84, 5.36]	0.44
MRF_u**	− 2.63 [− 2.81, − 2.47]	− 2.47 [− 2.56, − 2.26]	0.046
ARR_l	4.9 [4.42, 5.32]	4.75 [4.56, 5.21]	0.79
ARF_l	− 1.53 [− 1.65, − 1.41]	− 1.51 [− 1.62, − 1.37]	0.4
MRR_l	5.27 [4.77, 5.68]	5.12 [4.92, 5.58]	0.62
MRF_l**	− 2.6 [− 2.67, − 2.52]	− 2.27 [− 2.46, − 2.23]	0.008
∆ARR	− 0.22 [− 0.52, − 0.13]	− 0.04 [− 0.32, 0.13]	0.66
∆ARF	− 0.12 [− 0.31, − 0.02]	− 0.07 [− 0.16, 0.02]	0.99
∆MRR	− 0.22 [− 0.44, − 0.11]	− 0.05 [− 0.32, 0.17]	0.61
∆MRF	− 0.07 [− 0.2, 0.08]	− 0.04 [− 0.29, − 0.02]	0.93

This table compares values for COA s/p surgical repair and control subjects. Units for slope are normalized amplitude/time and values are reported as the median, 1st and 3rd quartiles of PPG features. Subscripts indicate upper (u) vs lower (l) extremity acquisition site. The ∆ indicates the difference between the upper and lower extremity for that feature. The two-sided Wilcoxon rank sum test was used to test for significance. Features with statistical significance between the 2 groups are marked as **p value < 0.05

*ARR* average rate of rise, *ARF* average rate of fall, *MRR* maximum rate of rise, *MRF* maximum rate of fall, *COA* coarctation of the aorta, *Q1* 1st quartile, *Q3* 3rd quartile

### Preoperative COA vs. postoperative COA

Table 4 shows the COA group compared to themselves preoperatively vs postoperatively relative to the COA repair.

**Table 4:** Preoperative vs postoperative waveform characteristics for differences between upper and lower extremities

Waveform features	Preoperative	Postoperative	*p* value
	Median [Q1, Q3]		
∆ARR	− 1.2 [− 4.5, 0.19]	− 0.16 [− 3.3, 2.6]	0.13
∆ARF	− 2 [− 2.6, − 0.085]	− 0.63 [− 1.7, 0.34]	0.2
∆MRR	− 1.2 [− 4.6, 0.24]	− 0.14 [− 3.3, 3]	0.16
∆MRF**	− 2.4 [− 3.8, 1.3]	− 0.3 [− 2.9, 0.71]	0.028

Reported as the median, 1st and 3rd quartiles of PPG features evaluated for COA before and after surgical repair. Subscripts indicate upper (u) vs lower (l) extremity acquisition site. The ∆ indicates the difference between the upper and lower extremity for that feature. The two-sided Wilcoxon rank sum test was used to test for significance. Features with statistical significance between the 2 groups are marked as **p value < 0.05

*ARR* average rate of rise, *ARF* average rate of fall, *MRR* maximum rate of rise, *MRF* maximum rate of fall, *COA* coarctation of the aorta, *Q1* 1st quartile, *Q3* 3rd quartile

### Leave-one-out cross-validation

**Fig. 5:**
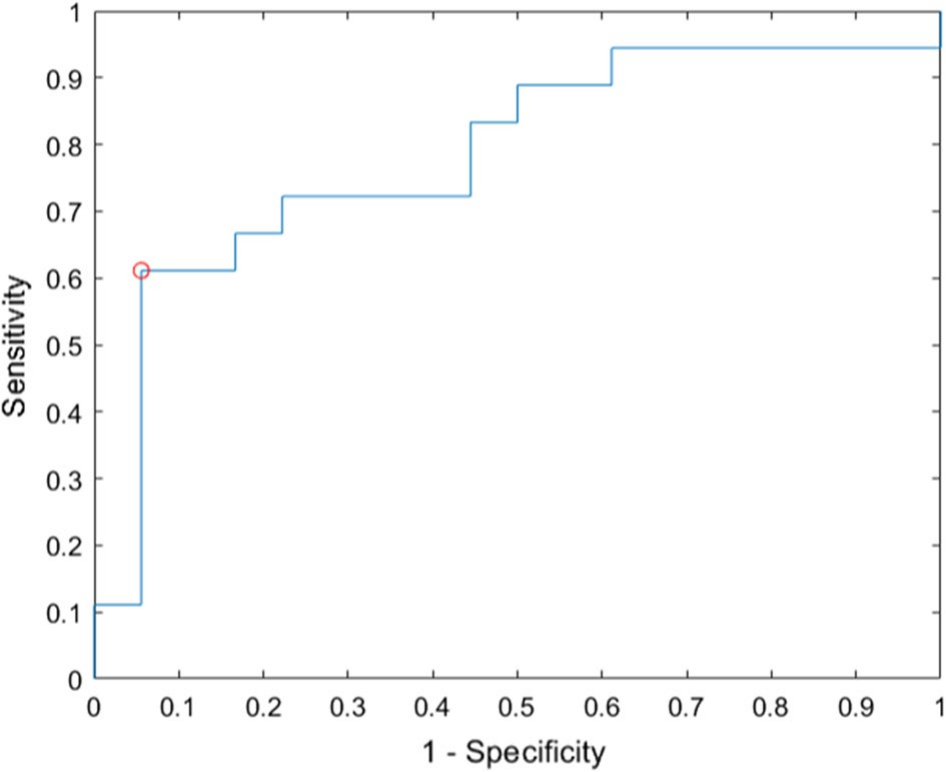
Utility for the proposed pulse oximetry screening algorithm. Here we show the receiver operating characteristic curve for the linear discriminant analysis classifier used in classifying subjects as having a COA or not. The area under the curve is 0.78. The suggested operating conditions are indicated by the red circle, with a suggested operating point at a sensitivity of 0.61 and a specificity of 0.94

In order to test the validity of our algorithm to identify and appropriately characterize patients with COA, we implemented leave-one-out cross-validation (LOOCV) stratified by patient as the number of subjects is too few to split the dataset into a train and test set. The area under receiver operating characteristic curve (AUROC) is generated for the LOOCV performance of the classifier. The MRF in the upper and lower extremity are used to classify subjects in the COA or control groups. A linear discriminant analysis (LDA) classifier achieved an average accuracy of 72%. The receiver operating characteristic curve (ROC) is given in Fig. 5. The area under the curve (AUROC) is 0.78. The suggested operating point is indicated by a red circle with a sensitivity of 0.61 and a specificity of 0.94, providing approximately double the sensitivity of detection of COA compared to current screening.

## Discussion

Our data show that it is indeed possible to correctly identify COA by identifying slope discrepancies of PPG waveforms between the upper and lower extremities of neonates. This was most notable when comparing the maximum rate of fall of the PPG signal in both the upper and lower extremities and obtaining the difference between them. In our data, the MRF remains significant in the lower extremity for the COA group when compared to controls and the ∆MRF does not, emphasizing the importance of needing an upper extremity as a reference for measuring the rate of flow in the lower extremity. This observed difference between COA and controls resolves following surgical correction suggesting that the fixed obstruction of COA and that the COA itself limits the maximum rate of blood flow in the lower extremities. This corresponds to a well-documented finding on echocardiographic assessment of coarctation of the aorta, known as diastolic tailing [12, 13]. When choosing a threshold for this new screening tool, a lower false-positive rate would be preferred, even if the sensitivity is sacrificed. Our data suggest that this detection algorithm could improve the sensitivity of screening from 40 to 60% which would be a significant improvement from the standard of care.

Our study is limited by its retrospective nature and small cohort of patients. Additionally, the signal analysis between upper and lower extremities did not occur simultaneously as was done in the studies of neonates with COA [9] and premature neonates with PDAs [11] with a custom-built photodetector for PPG analysis. Simultaneous acquisition reduces error creeping into the data as the clinical situation of a critically ill neonate fluctuates from moment to moment, especially in terms of heart rate and blood pressure. However, we attempted to eliminate these confounders in the data accordingly by normalizing for heart rate and timing changes. Our study may also be limited by the general practice that any neonate with suspected COA is started on a prostaglandin infusion prior to transfer to our quaternary children’s hospital to prevent the patent ductus arteriosus from closing as an attempt to prolong fetal circulation and buy time for further clinical evaluation. For this reason, we excluded infants from our control group that had a significant ductus arteriosus. All of our coarctation group had PDAs at the time of our study, and it is not yet determined what waveform differences there may be from the PDA in our dataset, although studies have shown promise in using PPG to detect the presence and closure of PDA [10]. Our study’s strength lies in the number of patients with usable waveforms obtained that had definitive COA requiring surgical repair in the neonatal period.

Future directions for this research include validating this algorithm with simultaneously acquired pulse oximetry waveforms from upper and lower extremities to eliminate signal noise acquired due to changing patient condition, validating the screening utility in a larger study in a newborn nursery among patients without suspected coarctation as well as exploring additional clinical conditions for this non-invasive screening technology. One potential area of additional clinical utility would be to screen for vascular anomalies (interrupted aortic arch, aberrant subclavian insertion, aortic dissection, aneurysm and perhaps even obstruction due to atherosclerosis or thrombosis) in a new way with pulse oximetry that is readily available at the patient bedside without waiting for advanced imaging.

## Conclusions

We identified specific differences in waveform characteristics of PPG signals in patients with COA using beat-by-beat analysis of multiple extremities in the same patients. This provides a valuable clinical application for data analytics in healthcare by comparing waveform morphology to improve the sensitivity of COA diagnosis. We hope to eventually implement this potentially life-saving screening method to identify neonates with COA early enough to prevent cardiogenic shock commonly associated with acute COA. Future directions include validating this screening method on a cohort of prospective, unscreened newborns.

## Methods

We retrospectively identified a cohort of neonates less than 30 days of age at time of admission to the cardiac intensive care unit who required surgical repair of COA over a 2-year period beginning in 10/2016 through 9/2018 at a quaternary children’s hospital. IRB approval was obtained for a retrospective chart review. Each of these patients was started on a prostaglandin infusion to maintain patency of the ductus arteriosus prior to surgery. These patients were matched 1:1 with controls who were the next closest admission meeting inclusion and exclusion parameters. Controls were excluded for age greater than 30 days of age at time of admission, with concurrent left heart obstructive lesions or a PDA. Single-ventricle patients and those with significant anatomic abnormality of the great vessels were also excluded.

PPG waveforms were digitally recorded from multiple patient sites at different times (upper and lower extremities) for the duration of their intensive care stay. Patients with inadequate recordings (due to poor signal quality or lack of recording) were omitted from analysis. The PPG waveform was recorded at least at 125 Hz to maintain waveform fidelity (waveforms that were recorded at 250 Hz were resampled at 125 Hz to maintain consistency and for ease of use due to file size). Nursing notes documented pulse oximetry probe acquisition site, and we allowed a 30-min grace period to minimize risk of any imprecise documentation of the timing of site change. If there was at least 1 h of data from a single acquisition site, that continuous waveform was saved for further analysis.

These signals were then processed through a bandpass filter (0.5–8 Hz) in order to remove baseline drift and high-frequency noise [14]. The continuous waveform for each site was traversed incrementally and analyzed in Matlab to find a 1-min segment that met criteria for a clean waveform defined as a signal quality index greater than or equal to 90% (as described in previously published work [15]). Clean segments were normalized by amplitude and by heart rate to eliminate confounders of different pulse oximetry acquisition with different devices and at different times. Each of the waveform characteristics was normalized by heart rate to reduce physiologic differences in patient condition from moment to moment.

**Fig. 6:**
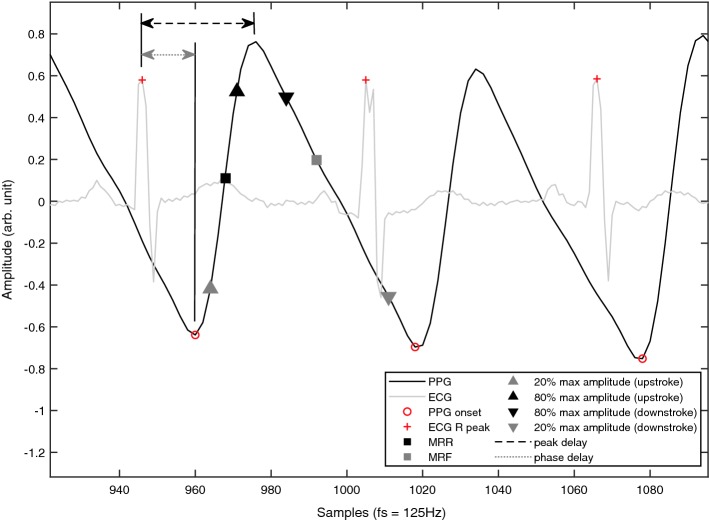
Schematic of waveform analysis. Here we demonstrate a pulse oximetry waveform with superimposed electrocardiogram (ECG). Rate of rise and fall are determined by specific points for each waveform cycle. The maximum (rate of rise or fall) is calculated over a rolling 40-ms window between the points at the trough and peak. The average (rate of rise or fall) is calculated between points located at the 20th and 80th percent of the waveform amplitude. Phase delay and peak delay are the time intervals between the peak R wave of ECG to the trough or peak of the pulse oximetry waveform as indicated

We evaluated four main waveform features which are illustrated in Fig. 6. The slope of a line connecting points at 20% and 80% of the max PPG wave amplitude on the upslope produced the average rate of rise (ARR) and on the down slope produced the average rate of fall (ARF). This slope is representative of the average rate of rise and fall of the blood flow rate based on the method demonstrated by Itu et al. in order to estimate the blood flow rate in the descending aorta [16]. Maximum rate of rise (MRR) and maximum rate of fall (MRF) were calculated over short 40-ms windows incrementally along the waveform and the greatest value was reported. This method allowed estimation of the max rate of rise while filtering against noise, compared to evaluation of the max rate from the first derivative of the PPG wave [17].

Waveform features for each site were computed by taking the median of all the indexed median values for that feature for each 1-min segment. This was repeated for each PPG site change of sufficient duration and signal quality (as defined above). We also calculated the difference between the upper and lower extremities to express the difference between those two site locations for each patient. In this way, we could evaluate the PPG waveform from extremities supplied by branch vessels both proximal and distal to the site of COA (see Fig. 2).

Each of these waveform features was evaluated for each patient’s PPG signal. Those resultant median wave features were analyzed in two main group analyses. First, we compared patients with pre-surgical COA to the unaffected controls, and second we compared pre-surgical COA to themselves as post-surgical COA following surgical repair. Statistical methods to evaluate the differences in waveform characteristics between groups utilized the two-sided Wilcoxon rank sum. We identified the features with the greatest differences that had statistically significant *p*-values (*p* < 0.05) as features that classify a PPG waveform as either belonging to a COA or control patient. Then, LOOCV was used to evaluate an LDA classifier trained on the resultant features that are useful in discriminating between the control and COA populations.

## Supplementary information

**Additional file 1: Figure S1.** Normalizing Slope by Heart Rate. Using a control patient, we observed a near linear association between the average rate of rise and fall compared to heart rate. The faster the heart rate the greater the slope of the rise and fall of the waveforms, in order to accommodate the rapid cardiac cycle. (A) Shows the strong negative correlation between heart rate and rate of fall. (B) Shows the strong positive correlation between heart rate and rate of rise.

## Data Availability

The data that support the findings of this study are available from Children’s Healthcare of Atlanta, but restrictions apply to the availability of these data, which were used under license for the current study, and so are not publicly available. Data are however available from the authors upon reasonable request and with permission of Children’s Healthcare of Atlanta.

## Abbreviations

COA: Coarctation of the aorta
CCHD: Critical congenital heart disease
PPG: Photoplethysmography
PDA: Patent ductus arteriosus
ARR: Average rate of rise
ARF: Average rate of fall
MRR: Maximum rate of rise
MRF: Maximum rate of fall
ECG: Electrocardiogram
LOOCV: Leave-one-out cross-validation
LDA: Linear discriminant analysis
Q1: 1st quartile
Q3: 3rd quartile
