# Supplementary figures and images for: Using pulse oximetry waveforms to detect coarctation of the aorta
Source: Biomed Eng Online. 2020 May 14;19:31. doi: 10.1186/s12938-020-00775-2 (PMC7227302; doi:10.1186/s12938-020-00775-2)

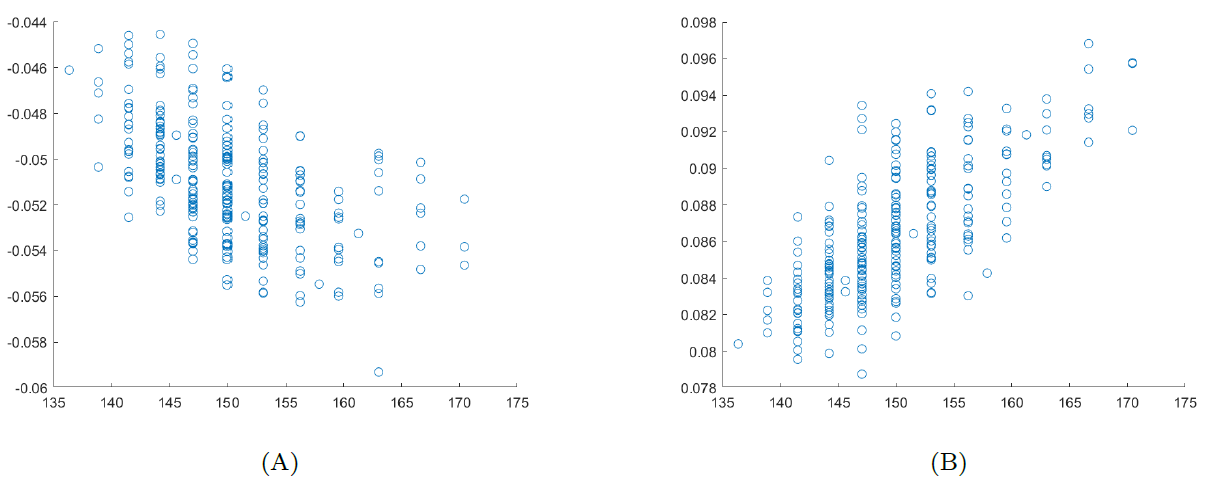

Supplement: Supplementary file 1 — Additional file 1: Figure S1. Normalizing Slope by Heart Rate. Using a control patient, we observed a near linear association between the average rate of rise and fall compared to heart rate. The faster the heart rate the greater the slope of the rise and fall of the waveforms, in order to accommodate the rapid cardiac cycle. (A) Shows the strong negative correlation between heart rate and rate of fall. (B) Shows the strong positive correlation between heart rate and rate of rise. [file 12938_2020_775_MOESM1_ESM.png]
